# Environmental and economic analysis of bioethanol production from sugarcane molasses and agave juice

**DOI:** 10.1007/s11356-021-15471-4

**Published:** 2021-07-24

**Authors:** Maria Magdalena Parascanu, Nestor Sanchez, Fabiola Sandoval-Salas, Carlos Mendez Carreto, Gabriela Soreanu, Luz Sanchez-Silva

**Affiliations:** 1 Department of Chemical Engineering, University of Castilla-La Mancha, Ciudad Real, Spain; 2 Energy, Materials and Environmental Laboratory, Department of Chemical and Biochemical Processes, Universidad de La Sabana, Campus Universitario Puente del Común, km. 7 Autopista Norte, Bogotá, Colombia; 3 Tecnologico Nacional de Mexico/ITS de Perote, Perote, Mexico; 4 Department of Environmental Engineering and Management, Technical University “Gheorghe Asachi” of Iasi, Iasi, Romania

**Keywords:** Bioethanol, Sugarcane bagasse, Agave bagasse, Life cycle assessment, Economic analysis

## Abstract

In this article, sugarcane molasses and agave juice were compared as potential feedstocks for producing bioethanol in Mexico in terms of their environmental impact and economic factors. Life cycle assessment (LCA) using SimaPro was carried out to calculate environmental impacts by using a cradle-to-gate approach. A preliminary economic analysis was performed to determine the economic feasibility of the studied options. Also, capital goods costs were obtained using the Aspen Plus economy package. Moreover, a sensitivity analysis was involved to compare the environmental and economic viability of producing bioethanol from sugarcane molasses and agave juice. LCA results revealed that cultivation and fermentation were the most harmful stages when producing bioethanol from sugarcane molasses and agave juice, respectively. Furthermore, when it was derived from agave juice rather than sugarcane molasses, it had more environmental benefits. This was ascribed to the lower consumption rate of fertilizers, pesticides, and emissions given off from the former. Regarding financial aspects, the preliminary analysis showed that producing bioethanol was not economically viable when grid energy alone was used. However, if power from the grid is partially replaced with renewable energy, producing bioethanol becomes economically feasible, and sugarcane molasses is the most suitable feedstock.

**Graphical abstract:**

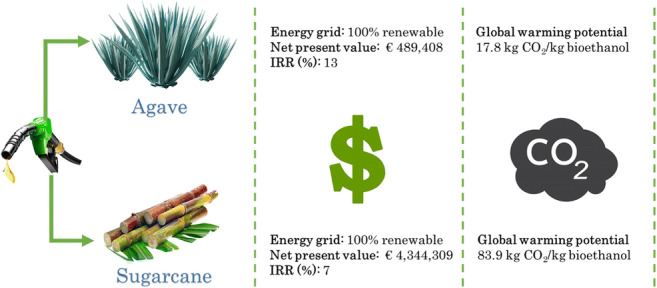

## Introduction

Undoubtedly, climate change—mainly due to the increase in CO_2_ emissions from fossil fuel combustion, industry, and transport—is a severe threat to life on our planet. For instance, about 3% of greenhouse gas (GHG) emissions are associated with transport (Oliver et al. 2017). Therefore, renewable energy poses an alternative for tackling these adverse effects (Sanchez et al. 2020b). Mexico, whose main source of energy is crude oil, is considered to be one of the largest contributors to CO_2_ emissions in Latin America (Hanif 2017; Sarmiento et al. 2019). Hence, it is generally agreed that it must change from crude oil to renewable fuels if it is to overcome the unfavorable effects of climate change (Rendon-Sagardi et al. 2014). Bioethanol is one potential renewable fuel, whose combustion is more efficient than gasoline, and, consequently, gives off fewer emissions of pollutants such as SOx, NOx, and particulate matter (Zabed et al. 2017).

Bioethanol is produced from a wide range of materials and can be classified into first, second, and third generation. First-generation bioethanol is produced from sugar and starchy feedstocks such as molasses and corn, while second and third generations are obtained from lignocellulosic materials and algae, respectively. Bioethanol production spans the following stages: physical pretreatment (i.e., crushing or chipping), hydrolysis (this is only required when both lignocellulosic and algae materials are employed as feedstock), fermentation, and distillation. For sugar materials, such as molasses, hydrolysis is not required since fermentable sugars, such as sucrose, glucose, and fructose, are freely available for metabolization by microorganisms during fermentation under anaerobic conditions. Yeasts, such as *Saccharomyces cerevisiae*¸ are the most widely used industrially, since they produce a large amount of ethanol and are highly tolerant to ethanol (Sanchez et al. 2020a; Sanchez et al. 2020c).

Sugarcane and agave are some potential feedstocks that could potentially be used in Mexico to produce bioethanol and mitigate the impacts associated with climate change. For instance, sugarcane (*Saccharum officinarum* L.) is an essential crop which is primarily used in sugar production. However, it has become fundamental for producing a wide range of goods in the industry. As a result, economic interests in this crop have increased significantly in recent years (Gómez-Merino et al. 2017; Lopez-Bustamante 2015). In Mexico, about 57 million tons of sugarcane is produced annually (SAGARPA 2018b). In the extraction process, by-products such as bagasse, sugarcane press mud, and molasses are also yielded (Dias et al. 2015). The latter is a by-product whose sugar content is 50%, which, in turn, is used to yield biopesticides, pharmaceuticals, cellulose, acids, and bioethanol, among other products.

Moreover, agave, also known as “*maguey*,” is a native crop from Mexico, and about 1.8 million tons of it are produced annually (SAGARPA 2018b). Nowadays, approximately 200 species are known, and they have been classified into three groups: wild, semi-cultivated, and cultivated (Mandujano Bueno et al. 2018; Nava-Cruz et al. 2015; Trejo-Salazar et al. 2016). Among these, *Agave salmiana* can grow in areas with low rainfall, low temperatures, and poor fertility soils; hence, it is considered to be economically viable. Furthermore, agave juice is well known for its ability to produce bioethanol by fermentation (Corbin et al. 2016; Tauer et al. 2004).

Although production is low in comparison to sugarcane molasses (1.8 million tons vs. 57 million tons), it has an outstanding economic, cultural, and social impact in Mexico (Pérez Hernández et al. 2016). Hence, it could potentially be used as a feedstock for producing bioethanol to mitigate GHG and to act as a driver for economic and social development in Mexico. Moreover, there is no land competition for food since agave grows on semiarid lands where food crops cannot be cultivated. Additionally, there is still enough unused land where agave can be cultivated. For instance, in Jalisco and Oaxaca, there are about 1.7 million and 60,000 hectares available respectively for cultivating agave, but at present it has only taken up 30% of this land (Núñez et al. 2011). In light of this, the environmental and economic benefits of the Mexican biofuel industry obtained from agave by-products were analyzed. This was performed by comparing it with a highly available feedstock such as sugarcane molasses. In order to assess the environmental benefits of agave crops, a life cycle assessment (LCA) was employed. This is an internationally standardized approach (International Organization for Standardization – ISO, i.e., ISO 14040 and ISO 14044) that enables environmental burdens associated with consuming resources and emissions to be assessed as well as the waste released in the chain of production (ISO14040 2006; ISO14044 2006).

To date, there are no studies in which the environmental impacts associated with bioethanol from both sugarcane molasses and agave juice are compared. However, several LCA studies on bioethanol yielded from both these raw materials have been published. For instance, Renouf et al. (2013) performed the LCA for ethanol production with different by-products from sugar extraction. They showed that sugarcane juice had the greatest impact on reducing nonrenewable energy and global warming potential (GWP). In addition, Silalertruksa et al. (2017) evaluated the environmental impacts from a sugarcane biorefinery, showing that this could be reduced by integrating waste valorization. Papong et al. (2017) studied the environmental benefits of producing bioethanol from cassava and molasses in Thailand, concluding that using it as a transport fuel reduced GHG emissions. However, eutrophication potential (EP) increased as did water consumption potential (WCP) in comparison with gasoline. Furthermore, Yan et al. (2011) evaluated bioethanol production from blue *Agave tequilana* Weber. They proved that agave was the optimum choice for producing first-generation bioethanol in comparison to corn, switchgrass, and sugarcane in terms of energy and GHG balances (Yan et al. 2011).

In short, since both crops were profitable in Mexico, it was deemed beneficial to determine which was most beneficial in terms of the environment and economy. In light of this, the goal of this study was to compare the environmental burdens and economic feasibility of producing bioethanol from sugarcane molasses and agave juice on the basis of these chains of production in Mexico.

## Methodology

### Life cycle assessment

#### Definition of goal and scope

A LCA was carried out considering the cradle-to-gate approach, in which the following stages were evaluated: (i) cultivation, (ii) juice extraction, (iii) fermentation, and (iv) distillation.

Bioethanol is characterized as being high in energy, 26.8 MJ/kg (Consorcio 2012; Ecoinvent 2019). For this reason, the production of bioethanol from molasses and agave juice to provide 1 MJ of energy was selected as the functional unit (FU) (Consorcio 2012). In this sense, considering the energy of bioethanol, to provide 1 MJ of energy, 3.73E-02 kg of bioethanol is needed.

#### System boundaries and assumptions

**Figure 1:**
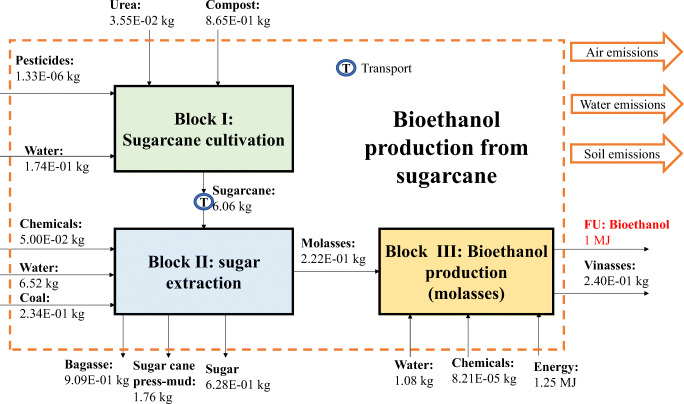
System boundaries for the bioethanol production, taking into account Block (I) sugarcane cultivation, (II) sugar extraction, and (III) sugarcane molasses fermentation

**Figure 2:**
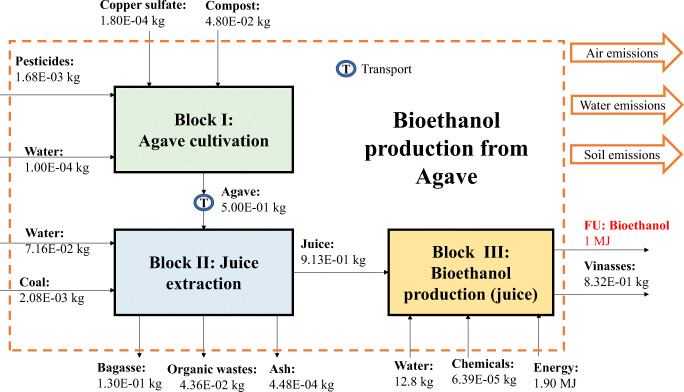
System boundaries for the bioethanol production, taking into account Block (I) agave cultivation, (II) agave juice extraction, and (III) agave juice fermentation

The LCA carried out for the bioethanol production system analyzed the entire chain of production, from cultivating sugarcane and agave to producing bioethanol from sugarcane molasses and agave juice. The main inputs in fermentation are generated at the extraction stage at which point molasses and agave juice were produced. Figures 1 and 2 represent the system boundaries considered for producing biofuels from sugarcane molasses and agave juice, respectively, considering the main inputs and outputs corresponding to each stage.

The following assumptions were made in this approach:

Chemical, fertilizer, pesticide, and energy production were included within the system boundaries as “market” dataset. A “market” dataset collects all activities with the same reference product in a certain geographical region, including the average amount of transport related to this product within that area (Ecoinvent 2019).

Transport of sugarcane and agave to the extraction plant were considered.

The plant extraction and the biorefinery plant were assumed to be in the same place.

Capital goods, staff, and buildings were excluded from this evaluation.

The system boundary excluded the usage and end of life for sugar and bioethanol products.

#### Life cycle inventory analysis

The primary inventory data for cultivating and extracting sugarcane and agave cultivation, sugar, and molasses/agave juice fermentation stages are shown in Tables 1–3, respectively.

**Table 1:** Inventory data for sugarcane and agave production stages (Block I, for FU=1MJ of bioethanol)

			Cultivation stage	
			Sugarcane	Agave
Input*				
Urea		kg	3.55E-02	-
Irrigation (river water)		m^3^	1.74E-01	-
Compost		kg	8.65E-01	4.80E-02
Allectus300sc (pyrethroid)		kg	7.39E-07	-
Engeo (pyrethroid)		kg	5.91E-07	-
Triple 17 (NPK)		kg	2.36E-02	-
Tap water		kg	8.26E-04	1.00E-04
Glyphosate		kg	-	1.80E-04
Copper sulfate		kg	-	1.80E-04
Bifentrina (pyrethroid)		kg	-	1.50E-03
Tillage (plowing)		ha	5.51E-05	-
Tillage (rolling)		ha	5.51E-05	-
Tillage (harrowing)		ha	5.51E-05	1.00E-05
Transport		kg*km	1.51E+02	2.55E+01
Output				
Sugarcane*		kg	6.06E+00	-
Agave*		kg	-	5.00E-01
Air emissions**	N_2_O	kg	1.94E-05	4.59E-04
	NH_3_	kg	2.94E-06	6.18E-05
	NO_x_	kg	4.07E-06	9.64E-05
	CO_2_	kg	9.79E-03	2.62E-01
	CH_4_	kg	3.29E-07	8.83E-06
Water emissions**	NO_3_	kg	5.00E-04	1.05E-02
	P_2_O_5_	kg	6.15E-06	1.45E-04
Organic waste*		kg	1.16E+01	5.00E-02

*from Mexico (real plant); **EPA (2016, 2017); Klein et al. (2006); Nemecek and Kägi (2007)

**Table 2:** Inventory data for sugar and agave juice extraction stages (Block II, for FU=1MJ of bioethanol)

		Extraction stage		Ref.
		Sugarcane	Agave	
Input				
Sugarcane	kg	6.06E+00	-	SAGARPA (2018a)
Agave	kg	-	5.00E-01	Livier (2004)
Flocculates	kg	7.15E-05	-	Consorcio (2012)
SO_2_	kg	6.06E-04	-	Consorcio (2012)
NaOH	kg	1.21E-03	-	Consorcio (2012)
Water	kg	6.52E+00	7.16E-01	Gamboa (2006); Livier (2004)
Quicklime	kg	4.85E-02	-	Consorcio (2012)
Coal	kg	2.34E-01	2.08E-03	Consorcio (2012)
Output				
Molasses	kg	2.22E-01	-	Consorcio (2012); Livier (2004)
Sugar	kg	6.82E-01	-	SAGARPA (2018a)
Agave juice	kg	-	9.13E-01	Livier (2004)
Bagasse	kg	9.09E-01	1.30E-01	SAGARPA (2018a)
Sugarcane press mud	kg	1.76E+00	-	Marín (2014)
Ash (waste)	kg	2.60E-02	4.48E-04	Consorcio (2012)
Organic waste	kg	-	4.36E-02	Livier (2004)
Air emissions				
H_2_O	kg	5.09E-06	7.09E-03	Livier (2004)
SO_2_	kg	6.45E-01	7.26E-07	Consorcio (2012)
CO_2_	kg	9.09E-05	9.21E-02	Consorcio (2012)
PM2.5	kg	1.82E-04	1.30E-05	Consorcio (2012)
NO_x_	kg	1.45E-05	2.59E-05	Consorcio (2012)
CO	kg	5.27E+00	2.07E-06	Consorcio (2012)
Heat	MJ	5.09E-06	7.52E-01	Consorcio (2012)

**Table 3:** Inputs and outputs of the bioethanol production from molasses and agave juice (Block III, for FU=1MJ of bioethanol) (from Aspen Plus® software)

		Biorefinery stage	
		Sugarcane	Agave
Input			
Molasses	kg	2.22E-01	-
Agave juice	kg	-	9.13E-01
Water	kg	3.36E-01	4.01E-01
Water (river)	m^3^	1.05E-02	1.24E-02
Energy	MJ	1.25E+00	1.90E+00
Urea^*^	kg	1.55E-05	-
MgSO_4_^*^	kg	6.66E-05	-
Ammonia sulfate^***^	kg	-	6.39E-05
Output			
Bioethanol	MJ	1.00E+00	1.00E+00
Vinasse	kg	2.40E-01	8.32E-01
Air emissions			
CO_2_	kg	4.33E-02	4.09E-02
Heat	kg	4.32E-02	4.36E-0-
Water emissions			
H_2_O	m^3^	1.02E-02	1.24E-02
Bioethanol	kg	3.77E-04	3.76E-02

*Pradeep and Reddy (2010); **Leaf (2017); ***López-Alvarez et al. (2012)

In this study, data collected for the raw material, utilities, and products at the cultivation stage were provided from a real plant in Mexico (Veracruz). However, air, water, and soil emissions at this stage were calculated according to the Intergovernmental Panel on Climate Change (IPCC), Environmental Protection Agency (EPA), and Ecoinvent (EPA 2016, 2017, Klein et al. 2006, Nemecek and Kägi 2007). In addition, the input and output data for the extraction stage were taken from the literature (Consorcio 2012; Gamboa 2006; Livier 2004; Marín 2014; SAGARPA 2018a). The mass and energy balances for the biorefinery plants were estimated by simulating the entire process with Aspen Plus® V.9 software (Aspentech, Bedford, MA, USA). Finally, the background processes were considered from the Ecoinvent database (Ecoinvent 2019).

## Block I: agriculture stage

### Sugarcane

In this study, a 5-year cycle was assumed for producing sugarcane. In the first year, the soil was prepared (by harrowing, plowing, and raking). Next, 20,000 kg/ha of compost was used for soil conditioning, which was transported 25 km from the “*La Gloria*” sugar refinery to the plot. Apart from compost, in order to make sugarcane productive, it is essential to use fertilizers and pesticides, as crop productivity depends on primary nutrients such as nitrogen, phosphorus, and potassium (Meyer 2013). To obtain the greatest yields from fertilizers, these should preferably be used when the soil is humid, as this helps in the dilution and absorption of nutrients (Meyer 2013). Specifically, in this study, fertilization was performed annually, and fertilizers and pesticides were transported 7 km in a 3-ton truck. The ones used were Triple17 (300 kg/ha), urea (150 kg/ha), Allectus 300sc (12 kg/ha), and Engeo (12 kg/ha).

Furthermore, the crop was irrigated with a gravity-fed system, using water from a river located 2 km away from the plot. Harvesting was performed manually, and the sugarcane was transported by truck to the mill, which was 25 km away. Total yields per annum were as follows: 1st year 140 tons/ha, 2nd year 120 tons/ha, 3rd year 100 tons/ha, 4th year 90 tons/ha, and 5th year 85 tons/year.

### Agave salmiana

*Agave salmiana* is used for producing alcoholic and nonalcoholic drinks. In this research, a 6-year cycle was assumed for agave cultivation. In the first year, the soil was prepared by harrowing. Planting was carried out in a rectangle (plants placed 3 m apart), which yielded an average of 1200 plants/ha.

The main advantage of using this plant is that it can be grown on highly degraded soils that are poor in nutrients and water (Davis et al. 2011). Pruning, which consisted in removing the outer leaves, which were already adult and dry, was carried out every two years. Here, fertilization was performed manually every year, with 4 tons/ha of compost made up of glyphosate (3 kg/ha), bifenthrin (20–30 kg/ha), and copper sulfate (3 kg/ha) during the rainy season. In addition, throughout the cultivation period, the crops were rain-fed only. Agave yielded 1200 plants/ha whose average weight was around 250 kg/plant.

## Block II: raw material processing stage

### Sugar extraction

After transporting the sugarcane to the sugar extraction plant, it was weighed and then stored in baskets (Consorcio 2012). The sorted sugarcane was then transported in a conveyor belt system to choppers whose blades were used for splitting it. Next, it was crushed in six mills with three or four maces to extract the juice (Consorcio 2012). Meanwhile, water was added to extract the sucrose contained in the fibrous material, and the juice and bagasse were obtained at this point. The latter was evacuated in the fourth mill (Consorcio 2012). In order to reduce costs and the environmental impact, 50% of the bagasse was used as a fuel for generating electricity (Consorcio 2012). The rest was used as a raw material in thermochemical processes.

Subsequently, the resulting juice was weighed to define the proportion of calcium oxide to be added, and this mixture was heated to 102–105 °C. Afterward, came clarification at which point the juice was purified, with all impurities removed in the form of insoluble calcium salts (Consorcio 2012). Sucrose was then recovered from these solid impurities by filtration, to obtain juice and a solid by-product (sugarcane press mud) which can be used as compost (Consorcio 2012; Sanchez et al. 2017).

The filtered juice, whose sugar content was about 14 wt.%, was subjected to evaporation in an evaporation train to remove any excess water and to gain 60 wt.% solids (syrup) (Consorcio 2012). This syrup was then crystallized in three tanks in a vacuum. The liquid and solid phases were next separated by centrifugation to yield sugar and molasses (Consorcio 2012).

### Agave juice extraction

On maturity, the agave plant was harvested by removing the leaves until the center of the plant (which is called the *pineapple*) was reached (L Gutiérrez Coronado et al. 2007). Firstly, this was cooked in an autoclave using pressurized saturated steam (Livier 2004). The cooking by-product (syrup) was then collected in a tank. Next, the cooked *pineapple* was ground to obtain cut agave and organic waste. The former was washed to extract the first syrup while the organic waste (wet bagasse) was sent to the second mill. The second and third milling were carried out under the same conditions as the first one in order to obtain syrup and bagasse (Livier 2004). The three syrups obtained were called agave juice, which were then stored in a tank and fermented to obtain bioethanol. At this extraction stage, 50% of the resulting bagasse and 10 kg of coal were used to produce the electricity needed (Consorcio 2012).

## Block III: biorefinery plants

In this paper, bioethanol produced from molasses and agave juice was yielded at various stages. During fermentation (first stage), microorganisms, the most commonly used of which were yeasts (e.g*.*, *S. cerevisiae*) (Robak and Balcerek 2018), converted sugars (glucose and fructose) into bioethanol and CO_2_ (Eq. 1) (Lin and Tanaka 2006).

1 $$ {\mathrm{C}}_6{\mathrm{H}}_{12}{\mathrm{O}}_6\to 2{\mathrm{C}}_2{\mathrm{H}}_5\mathrm{OH}+2\mathrm{C}{\mathrm{O}}_2 $$

Distillation was the second stage and the aim of which was to obtain anhydrous bioethanol concentrated up to approximately 96%. The drawback to this was the large amount of energy used (Gavahian et al. 2016).

The final stage was dehydration in which anhydrous ethanol (i.e., 99.7 wt. %) was obtained by using molecular sieves (Robak and Balcerek 2018, Soreanu et al. 2004).

In this study, bioethanol production was simulated in Aspen Plus and using the non-random two-liquid (NRTL) method. Table 4 shows the features of both the sugarcane molasses and agave juice employed in this study.

**Table 4:** Characterization of the sugarcane molasses and agave juice (experimental data)

	Molasses (wt. %)	Agave juice (wt. %)
Water	49.70	89.80
Fatty acids	0.23	0.42
Sucrose	48.70	9.72
Lignin	0.25	0.02
Ash	1.12	0.04

**Figure 3:**
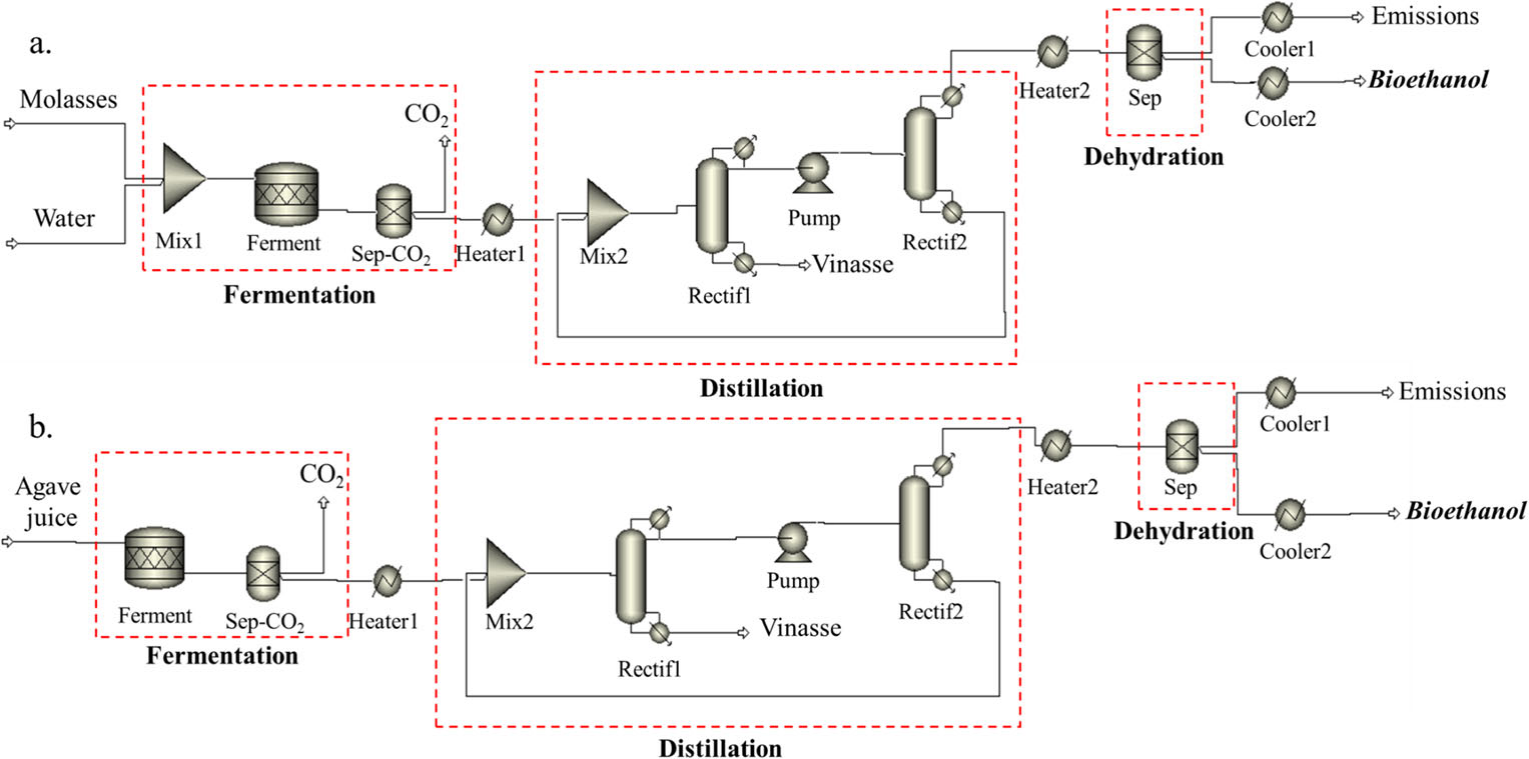
Aspen Plus® flow sheet simulation for the bioethanol production from: **a** sugarcane molasses and **b** agave juice

Table 5 gives a brief explanation of each block used for simulating bioethanol production. The flow sheet diagrams for obtaining bioethanol from sugarcane molasses and agave juice are shown in Figure 3.

**Table 5:** Block description for bioethanol production simulation

**Name**	**Equipment**	**Description**
Mix1 *	Mixer	To mix water with the raw material ^(1)^
**Ferment**	Rstoic	To convert the raw material into ethanol. It includes the fermentation stage
**Sep-CO**_**2**_	Sep	To separate the CO_2_ from the mainstream
**Heater1**	Heater	To heat the mainstream
**Mix2**	Mixer	To mix the mainstream with the by-product that results of the second rectification column
**Rectif1**	RadFrac	To concentrate bioethanol up to 50 % (w/w) ^(1)^ and 45 % (w/w) ^(2)^, respectively
**Pump**	Pump	To increase the mainstream pressure
**Rectif2**	RadFrac	To concentrate bioethanol up to 94 % (w/w)
**Heater2**	Heater	To heat the mainstream
**Sep**	Sep	To purify the mainstream and obtain 99.7 % (w/w) bioethanol
**Cooler1**	Heater	To cool the emission stream
**Cooler2**	Heater	To cool the bioethanol stream

*only molasses, (1) molasses, (2) agave juice

The difference between simulations was water requirements. This must be added to prevent yeast cells dying on account of the high osmotic pressure of the fermentation culture (Jambo et al. 2016). Indeed, sugarcane molasses, whose sugar concentration was 48.7 wt.% (Table 4), needs to be diluted until 30 wt.% is reached, while agave juice does not as it is lower in sugars (i.e., 9.8 wt.%).

Fermentation was the first stage and was simulated by means of a RSTOIC at 30 °C. In the fermenter, sucrose was converted to ethanol to obtain 14 wt.% and 4.7 wt.% ethanol for molasses and agave juice, respectively. In this study, it was assumed that sucrose was converted into glucose and fructose at a rate of 100%, while the rate for converting glucose and fructose into bioethanol and CO_2_ was assumed to be 85.7% (Ghani and Gheewala 2018).

The resulting CO_2_ was removed in Sep-CO_2_ equipment, while the remaining stream was heated to 85 °C. After heating, distillation was performed with two rectification columns (Rectif1 and Rectif2). In the former, 15 stages were employed, while the latter used 50. Feeding for the first column occurred at the 6th stage, while for the second column, it was the 49th. From the first column, bioethanol was obtained with 50 wt.% and 45 wt.% for molasses and agave juice, respectively. In the second column, the bioethanol was purified at 94 wt.%, a value close to that for azeotropic bioethanol (95.6 %) (Valencia and Cardona 2014). The by-product obtained in the first distillation unit (vinasse) was considered to be an avoided product.

The distilled stream was heated to 115 °C and introduced into the dehydration zone, which is commonly carried out with molecular sieves. In this study, these were modeled as a separator column. The resulting stream (i.e., 99.9 wt.% ethanol) was cooled (Cooler2) to 50 °C, whereas the output streams (i.e., emissions, water, and ethanol) were cooled (Cooler1) to 70 °C. Moreover, steam and cooling water were employed as the heat source for both distillation columns. In this study, steam was obtained by a water heater, while river water was used for cooling.

### Impact assessment methodology

The LCA was carried out using the SimaPro 8 software, with the ReCiPe 2016 midpoint (H) methodology to calculate the LCA results. The following impact categories were selected for determining the environmental performance of the bioethanol produced: GWP, ozone depletion potential (ODP), photochemical oxidation formation potential—humans (HOFP), photochemical oxidation formation potential—ecosystems (EOFP), terrestrial acidification potential (TAP), freshwater eutrophication potential (FEP), human toxicity potential—cancer (HTPc), human toxicity potential—non-cancer (HTPnc), fossil fuel potential (FFP), and WCP.

In the chain of production for bioethanol, different by-products were obtained. Therefore, economic allocations were used for the environmental burdens of co-products (Ecoinvent 2019). The economic allocation factors were as follows:

Sugar extraction: 80.6 % (0.58 €/kg) for sugar, 8.6 % (0.19 €/kg) for sugarcane molasses, 8.95 % (0.025 €/kg) for sugarcane press mud, and 1.85 % (0.01 €/kg).

Juice extraction: 99.3 % (0.2 €/kg) for agave juice and 0.7 % (0.01 €/kg).

Bioethanol production (molasses): 82.4 % (0.75 €/kg) and 17.6 % (0.025 €/kg).

Bioethanol production (molasses): 57.4 % (0.75 €/kg) and 42.6 % (0.025 €/kg).

### Preliminary cost analysis

A preliminary cost analysis was carried out to determine the economic feasibility of producing bioethanol from sugarcane molasses and agave juice. An economic evaluation was made using the percentage methodology (Hillstrom and Hillstrom 2002, Peters et al. 2003). The Aspen Plus® software was used for assessing the financial aspects related to equipment costs. In addition, the price of the storage tank was calculated according to its scale (Kalk and Langlykke 1986).The evaluation corresponded to V class evaluation economy. This approach is commonly used for screening alternatives, and all cost estimations were accurate between 30% and 50% (Becerra et al. 2017; Proaño et al. 2020).

Furthermore, by observing the quantity of utilities needed in the process, water and energy costs could be estimated. The sale price of the products (bioethanol and vinasse) also had to be set. The financial indicators considered in this study were the following: net present value (NPV), internal rate of return (IRR), and payback.

## Results

In this research, an environmental and economic analysis was performed to determine the most suitable crop for producing bioethanol. In this study, sugarcane molasses and agave juice were used as feedstock. The stages involved in converting these to bioethanol as well as the scenarios overall were compared. The conversion stages included cultivation, extraction, and biorefining. Moreover, an economic and sensitivity analysis of the bioethanol production stage was made to determine which of the two crops was more economically viable. In the following section, the environmental impacts for both feedstocks are shown.

### Producing bioethanol from sugarcane molasses

**Figure 4:**
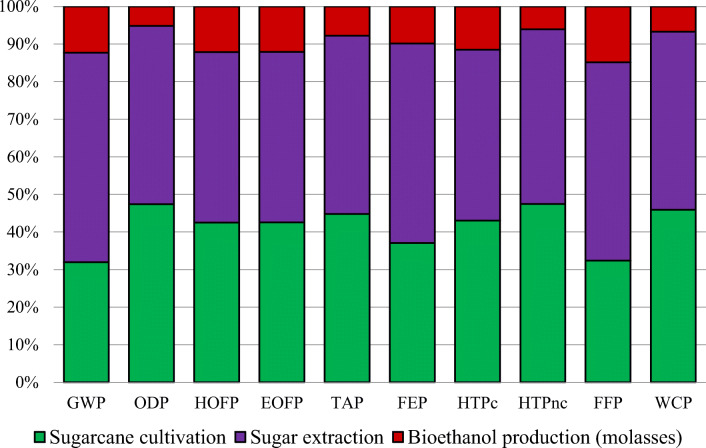
Characterized data for bioethanol production from sugarcane molasses, considering the cultivation, the sugar extraction, and the bioethanol production stages

In this section, the results of the “cradle-to-gate” analysis for producing bioethanol from sugarcane molasses are shown in Figure 4. In addition, the LCA results for each analyzed stage are presented in Table 6.

**Table 6:** Characterized results for bioethanol production from sugarcane molasses, considering all the three stages

Impact category	Unit	Sugarcane cultivation	Sugar extraction	Bioethanol production (molasses)
GWP	kg CO_2_ eq	1.04E+00	1.82E+00	3.99E-01
OPD	kg CFC11 eq	1.20E-05	1.20E-05	1.31E-06
HOFP	kg NO_x_ eq	1.69E-03	1.80E-03	4.83E-04
EOFP	kg NO_x_ eq	1.73E-03	1.85E-03	4.92E-04
TAP	kg SO_2_ eq	8.27E-03	8.75E-03	1.43E-03
FEP	kg P eq	6.55E-05	9.38E-05	1.74E-05
HTPc	kg 1,4-DCB eq	2.23E-03	2.36E-03	5.95E-04
HTPnc	kg 1,4-DCB eq	1.16E-01	1.13E-01	1.49E-02
FFP	kg oil eq	1.96E-01	3.19E-01	8.97E-02
WCP	m^3^	1.99E-01	2.06E-01	2.90E-02

According to Figure 4, sugarcane extraction showed the greatest results in almost all impact categories whose values were higher than 45%. It had the highest impact on HTPnc (47%), whereas bioethanol production showed the lowest contribution in all categories (<15%). Concerning GWP, significant differences were observed among stages according to Table 6. Thus, sugar extraction had the highest impact value (1.82 kg CO_2_ eq) followed by sugarcane cultivation (1.04 kg CO_2_ eq) and bioethanol production (3.99E-01 kg CO_2_ eq). The results obtained for the former were mainly due to the high amounts of CO_2_ given off (Table 2) and background processes (quicklime and coal production). Also, the GWP impact value obtained for sugarcane cultivation was associated with the GHG given off and the diesel used in transport (Table 1).

Like GWP, FEP and FFP showed the same tendency. In this respect, the values obtained at sugar extraction in terms of FEP and FFP were 9.38E-05 kg P eq and 3.19E -01 kg of oil eq, respectively. The SimaPro software identified that the main contributing factors to FEP at the second stage were background processes, such as coal production and emissions during these processes. In terms of sugar extraction, using and producing coal for obtaining energy and steam were found to be the factors which had most impact on FFP. Moreover, the negative impacts on both categories were also due to P_2_O_5_ emissions, the use of diesel, P-based fertilizers, and compost (Table 1).

The high environmental impact on cultivating sugarcane was due to emissions from organic and inorganic fertilizers, water, and the diesel used. In ODP, the most influential factors were N_2_O emissions from N-based fertilizers and compost and the CH_4_ given off from transport from burning diesel (Table 1) (Papong et al. 2017). For HOFP and EOFP, the impacts with sugarcane cultivation (Table 6; 1.80E-03 and 1.85E-03 kg NO_x_ eq, respectively) were associated with NOx emissions from transport and background processes (energy and diesel production) (Table 1). NH_3_ and NO_x_ emissions from cultivation (transport and using fertilizer and pesticide) were the main contributors to TAP. In addition, SO_x_ emissions from fertilizers and energy production (background processes) significantly contributed to this. Also, higher values were observed for HTPnc than those for HTPc for sugarcane cultivation (Table 6). According to SimaPro’s data, these impacts were mainly associated with background processes (fertilizer and pesticide production) and emissions (e.g., benzene, cadmium, nickel, chromium) (Silalertruksa et al. 2017). Finally, WCP was affected by the high amounts of water used in irrigation and preparing fertilizers (Table 1).

### Producing bioethanol from agave juice

**Figure 5:**
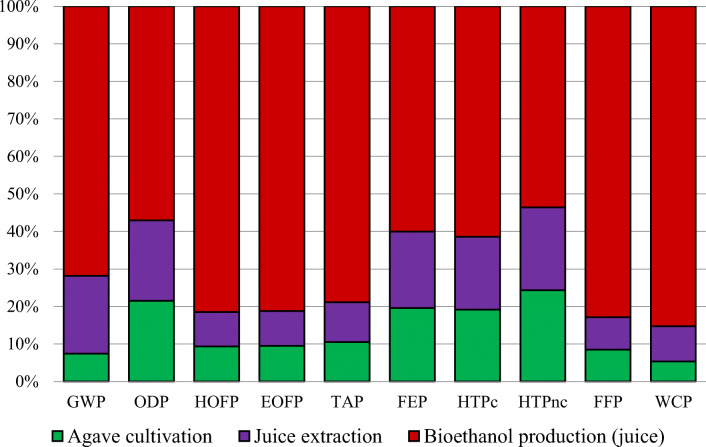
Characterized data for bioethanol production from agave juice, considering the agave cultivation, agave juice extraction, and bioethanol production stages

Figure 5 shows the results for the agave-to-bioethanol chain, considering the ten selected categories. Table 7 presents the LCA results for each stage under consideration in this research. All the impact values at each stage were calculated for 1 MJ of bioethanol produced.

**Table 7:** Characterized results for bioethanol production from agave juice, considering all the three stages

Impact category	Unit	Agave cultivation	Juice extraction	Bioethanol production (juice)
GWP	kg CO_2_ eq	4.99E-02	1.40E-01	4.83E-01
OPD	kg CFC11 eq	2.51E-07	2.50E-07	6.66E-07
HOFP	kg NO_x_ eq	6.42E-05	6.34E-05	5.60E-04
EOFP	kg NO_x_ eq	6.65E-05	6.56E-05	5.71E-04
TAP	kg SO_2_ eq	1.56E-04	1.58E-04	1.17E-03
FEP	kg P eq	7.01E-06	7.31E-06	2.15E-05
HTPc	kg 1.4-DCB eq	2.70E-04	2.74E-04	8.65E-04
HTPnc	kg 1.4-DCB eq	6.95E-03	6.33E-03	1.53E-02
FFP	kg oil eq	1.08E-02	1.10E-02	1.05E-01
WCP	m^3^	9.48E-04	1.66E-03	1.51E-02

According to Figure 5, bioethanol production contributed to the highest impact in all categories. The values obtained for this were the following: 72% (GWP), 57% (ODP), 81% (HOFP and EOFP), 79% (TAP), 60% (FEP), 61% (HTPc), 54% (HTPnc), 83% (FFP), and 85% (WCP). Additionally, both cultivation and juice extraction showed similar values in all categories, as shown in Figure 5.

As for agave, bioethanol production was the most environmentally damaging stage. This was associated with the low sucrose concentration and consequently low ethanol yield during fermentation, factors which affected performance. Therefore, a higher amount of both raw materials and utilities was required to produce 1 MJ of bioethanol from agave juice in comparison to sugarcane molasses.

The information generated by SimaPro software indicated that producing and using grid energy to produce bioethanol were the main explanatory factors behind this detrimental environmental impact (Table 3). Energy production, considered to be a background process, had a significant influence on almost all the categories analyzed (GWP, ODP, HOFP, EOFP, TAP, HTPc, HTPnc, and FFP), mainly due to the large amount of emissions. For instance, NO_x_ emissions were observed to be primarily responsible for the values obtained in HOFP, EOFP, and TAP. Also, CH_4_ emissions (background processes) were detrimental to GWP and ODP (Nguyen and Gheewala 2008, Zhang et al. 2010). The high value of GWP (6.72E-01 kg CO_2_ eq) was also due to the CO_2_ given off when fermenting agave juice (Table 3) (Amores et al. 2013; González-García et al. 2012; Wang et al. 2013). Human toxicity categories were affected by emissions such as those from nickel, cadmium, chromium, and formaldehyde that were given off mainly in energy and chemical production. Raw materials such as coal, natural gas, and oil used in background processes were found to be the main components which influenced FFP. Also, the effect producing bioethanol had on FEP was related to agave cultivation and juice extraction, while WCP was affected by the water consumed at the last stage (Table 3).

At the cultivation stage, using fertilizers and transport had a high impact on ODP due to CH_4_ and N_2_O emissions. According to Table 7, the impact values for HOFP and EOFP were 6.42E-05 and 6.65E-05 kg NO_x_ eq, respectively, and these were attributed to NO_x_ emissions (Table 1) given off when raw materials, fertilizers, and pesticides were being transported. Transportation, using fertilizers, and compost made a significant contribution to TAP as they generated high amounts of NO_x_ and NH_3_ (Table 1). In addition, background processes such as producing fertilizers and pesticides were harmful in terms of HTPc and HTPnc (Silalertruksa et al. 2017).

### Producing bioethanol from molasses vs. agave juice

**Figure 6:**
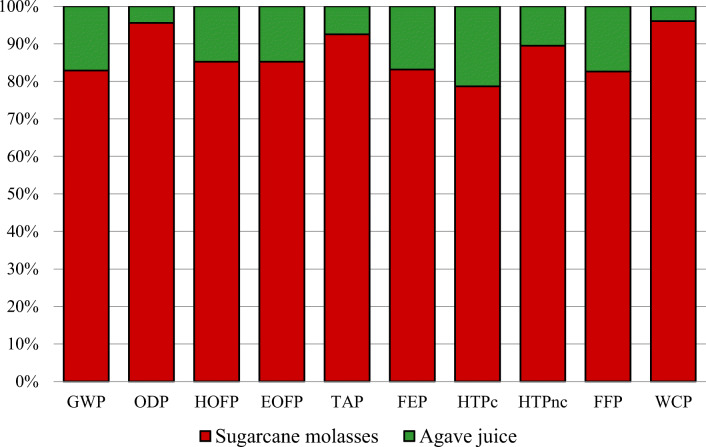
Relative environmental impacts for bioethanol production from sugarcane molasses and agave juice

Figure 6 compares the relative environmental impacts for producing bioethanol from sugarcane molasses and agave juice. Table 8 shows the impact values for 1 MJ of bioethanol produced from sugarcane molasses and agave juice.

**Table 8:** LCA analysis comparing the production of 1 kg of bioethanol from sugarcane molasses and agave juice

Impact category	Unit	Sugarcane molasses	Agave juice
GWP	kg CO_2_ eq	3.26E+00	6.72E-01
OPD	kg CFC11 eq	2.53E-05	1.17E-06
HOFP	kg NO_x_ eq	3.97E-03	6.88E-04
EOFP	kg NO_x_ eq	4.07E-03	7.03E-04
TAP	kg SO_2_ eq	1.85E-02	1.48E-03
FEP	kg P eq	1.77E-04	3.58E-05
HTPc	kg 1.4-DCB eq	5.19E-03	1.41E-03
HTPnc	kg 1.4-DCB eq	2.44E-01	2.86E-02
FFP	kg oil eq	6.04E-01	1.27E-01
WCP	m^3^	4.33E-01	1.77E-02

On comparing both scenarios, bioethanol produced from agave juice was seen to make a relatively minor contribution in all categories. However, in the previous analyses, impacts on producing bioethanol from agave juice were observed to be higher than those for molasses. Hence, agave juice is more environmental-friendly. This significant difference could be due to the different ways these raw materials are cultivated and processed. Therefore, in this way, molasses was seen to generate much higher impact values than agave juice (Tables 6 and 7), and, consequently, molasses were more harmful to the environment overall.

When converting sugarcane-to-bioethanol, the amount of GHG emissions was 384% higher than those for agave-to-bioethanol. Indeed, GHG for sugarcane was 3.26 kg of CO_2_-eq/MJ, while for agave; this figure was only 0.67 kg. GHG emissions, as well as using N-fertilizers, coal, and energy, increased the value of GWP (Nguyen and Gheewala 2008, Pryor et al. 2017, Wang et al. 2012). In addition to CH_4_ and N_2_O, the data provided by SimaPro indicated that emissions of Halon-1211, Halon-1301, CFC-10, and CFC-12 were the most detrimental to the environment in terms of ODP (González-García et al. 2012). Also, the impact value obtained in this category could be linked to cultivation. At this point, pesticides (which may contain CH_4_ and halocarbon compounds) were used. In Table 8, it was observed that the impact value for ODP in molasses was higher than that in agave juice. This may be because more pesticides were required, and more gases were given off to cultivate sugarcane than agave (Table 1).

According to Tables 1 and 2, global NO_x_ emissions in sugarcane-to-bioethanol were 1.86E-05 kg/MJ of bioethanol, while in agave-to-bioethanol, they were 1.22E-04 kg/MJ of bioethanol, respectively. Moreover, NO_x_, SO_x_, NH_3_, CO, and hydrocarbons were given off on producing and using fertilizers and pesticides, transport, burning coal and bagasse, and energy production were the main contributing factors to the following: HOFP, EOFP, and TAP, as shown in Table 8 (Brizmohun et al. 2015, Costa et al. 2018, Ghani and Gheewala 2018, Ruiz et al. 2018).The higher amount of NO_x_ given off and greater consumption of these feedstocks (i.e., coal, pesticides, and fertilizer) in sugarcane meant that bioethanol from this raw material had a greater environmental impact in terms of HOFP, EOFP, and TAP than agave (Figure 6 and Table 8) (Brizmohun et al. 2015; Michailos 2018)

Figure 6 and Table 8 show that in terms of human toxicity, values for sugarcane were up to approximately 70% higher than they were for agave (78% for HTPc and 89% for HTPnc). This may be because sugarcane is relatively more reliant on fertilizers, pesticides, coal, and diesel than agave. It was also on account of the high emissions given off with the former (Tables 1–3) (Ghani and Gheewala 2018, Han et al. 2019, Ruiz et al. 2018). Moreover, on producing energy, fertilizers, pesticides, chemicals, diesel, coal, and compost (background processes), pollutants such as nickel, cadmium, chromium, and formaldehyde (that damaged the environment in terms of HTPc and HTPnc) were given off (Brizmohun et al. 2015).

In sugarcane cultivation, considerably more fertilizers, pesticides, and compost were used. Additionally, this process created the highest amount of wastewater ash and emissions (P_2_O_5_) (Table 1) all of which led to a greater impact on FEP than agave did (Figure 6 and Table 8) (Brizmohun et al. 2015, Costa et al. 2018, Ghani and Gheewala 2018, Michailos 2018, Ruiz et al. 2018).

Finally, the raw materials used (coal, natural gas, and oil) for producing diesel and chemical products were the main contributing factors to FFP (Table 8) (Brizmohun et al. 2015, Ghani and Gheewala 2018). Moreover, the water used in irrigation (sugarcane), preparing fertilizers and pesticides, extracting sugar and agave, and producing bioethanol contributed to WCP (Table 8) (Papong et al. 2017). As observed in the other categories, as well as FFP and WCP, sugarcane had higher impact values than agave (Figure 6).

### Recommendations for improving environmental performance

Several recommendations for making bioethanol from sugarcane molasses and agave juice more environmentally friendly could be considered. One of the greatest challenges to meet is making the raw material more productive without damaging the ecosystem (Farahani and Asoodar 2017, Osei et al. 2003, Papong et al. 2017, Silalertruksa and Gheewala 2009, Steiner et al. 2007).

In this respect, soil quality must be improved by substituting inorganic fertilizers with organic ones, such as manure or compost (Osei et al. 2003; Steiner et al. 2007). Also, this would considerably reduce eutrophication (Silalertruksa and Gheewala 2009). Similarly, reducing organic waste and emissions into the atmosphere also improves the environmental performance at the cultivation stage. A decrease in CH_4_, CO_2_, N_2_O, and NO_x_ emissions in turn reduces impact values in terms of GWP, HOFP, EOFP, and TAP, among others (Silalertruksa and Gheewala 2009).

For sugar and agave juice extraction, coal-produced energy was primarily responsible for the negative environmental impact. In this respect, it is recommended substituting coal with another fuel or using renewable energy such as biomass or hydraulic energy (the most widespread in Veracruz, Mexico) (CEMAD 2016, Farahani and Asoodar 2017) as this reduces GHG emissions and environmental damage in sugar extraction.

**Figure 7:**
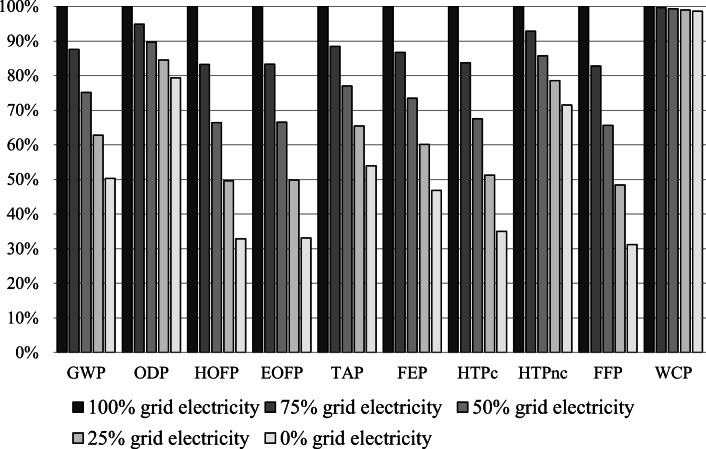
Sensitivity analysis for the bioethanol production from sugarcane molasses

**Figure 8:**
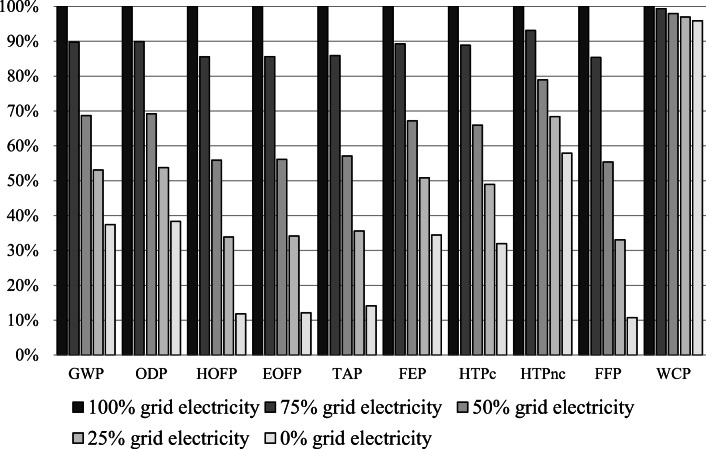
Sensitivity analysis for the bioethanol production form agave juice

Finally, to reduce the impact that bioethanol production has on the environment, the amount of grid energy consumed must be reduced. In this respect, as in the processing stage, it is recommended replacing grid energy with that generated from renewable sources (biomass or hydraulic). Using renewable energy at the ethanol production stage could help reduce GHG emissions. In this sense, a sensitivity analysis was carried out in which grid energy was increasingly replaced with renewable energy. The main results of these analyses can be seen in Figures 7 and 8 and in Table 9. The sensitivity scenarios are as follows:

**Table 9:** The equipment and utilities costs for bioethanol production from molasses and agave juice

Equipment costs			
		Sugarcane molasses	Agave juice
Storage tank	€	35,000	36,000
Fermenter	€	38,000	37,900
Heater 1	€	7100	7100
Rectifier 1	€	66,040	66,950
Pump 1×2	€	7200	7200
Rectifier 2	€	33,400	34,000
Heater 2 × 2	€	9700	9700
Separator	€	13,700	13,700
Cooler 1	€	5400	5100
Cooler 2	€	7500	7100
Major purchased equipment (E)	€	208,400	200,100
Utility costs			
Energy	€/year	768,000	288,000
Water	€/year	7200	2000
Yeast	€/year	46,400	30,400
Urea/ammonia sulfate	€/year	200	100
MgSO_4_	€/year	13,200	-
Total utilities costs	€/year	829,000	320,500

Base scenario: 100% grid energy and 0% renewable energy

Scenario 1: 75% grid energy and 25% renewable energy

Scenario 2: 50% grid energy and 50% renewable energy

Scenario 3: 25% grid energy and 75% renewable energy

Scenario 4: 0% grid energy and 100% renewable energy

Figures 7 and 8 show that by changing from the Mexican energy grid to renewables, most of these impacts will be significantly reduced. In this research, we assumed that renewable energy would not have an environmental impact. For instance, GWP would be reduced by almost 50%, if the energy came from renewable sources and sugarcane was employed to produce bioethanol. This reduction was based on the fact that the energy grid in Mexico was mainly oil-based (>60%), while renewables still accounted for under 20% (Sarmiento et al. 2019). A reduction in oil consumption would cause a fall in GHG emissions. However, a higher drop would be observed if agave was employed as the feedstock. The relatively higher drop for agave was associated with the energy consumption required to produce bioethanol. According to Figures 1 and 2, producing 1 MJ of ethanol from sugarcane and agave would require 1.25 and 1.90 MJ of energy, respectively.

Apart from this strategy, using vinasse as compost may significantly reduce environmental damage. It is also essential to capture and store any CO_2_ given off on producing bioethanol by means of carbon capture and storage technology (CEMAD 2016, Farahani and Asoodar 2017, Laude et al. 2011, Silalertruksa and Gheewala 2009).

### Comparison with other studies

As earlier mentioned, there is little research on producing bioethanol from agave (Yan et al. 2011). However, several articles concerning the environmental screening of bioethanol produced from sugarcane have been published. For instance, Farahani and Asoodar (2017) reported that sugarcane cultivation mainly contributed to acidification, ozone layer depletion, human toxicity, and photochemical oxidation. In addition, sugar extraction mainly contributed to global warming potential. Moreover, Amores et al. (2013) demonstrated that sugarcane cultivation is the main hotspot in the life cycle since it affected almost all categories except eutrophication. Similarly, Silalertruksa and Gheewala (2009) observed that it was the main contributing factor to the environmental impact in terms of global warming, photooxidation, acidification, human toxicity, and eutrophication.

As observed in this study, cultivation was not the main hotspot when producing bioethanol from sugarcane. In this paper, sugar extraction contributed to a greater extent of the environmental impact than cultivation and bioethanol production. Indeed, it accounted for at least 46% in all the categories assessed.

According to the literature review, global warming potential ranged between 0.016 and 400 kg CO_2_ produced for 1 MJ of ethanol from sugarcane (Amores et al. 2013, Farahani and Asoodar 2017, Silalertruksa and Gheewala 2009, Valencia and Cardona 2014). Table 8 shows that around 3.26 kg CO_2_-eq/MJ was given off when sugarcane molasses was the feedstock. In other words, it can be concluded that the observed carbon footprint is quite similar for that previously reported in other research. These discrepancies in the research were ascribed to (i) assessment models (e.g., CML and ReCiPe), (ii) allocation method, (iii) and inventory data.

Furthermore, when comparing the actual study with that of Ghani and Gheewala (2018), some similarities can be observed. They studied four different scenarios for producing bioethanol from molasses, the first of which was based on very similar assumptions to those we made. Thus, they considered using inorganic fertilizers and freshwater irrigation for cultivation, bagasse, and biogas (from treated wastewater from the bioethanol plant) to produce electricity. Cane waste was burned, wastewater was discharged into surface water, and filter cake was used as fertilizer. As in this study, they used the ReCiPe 2016 midpoint methodology and the SimaPro 8.4 software to evaluate impacts. On comparing the results obtained for the five categories in this research and those by Ghani and Gheewala (2018), similar values were observed in three of them (GWP, FEP, and FFP). The differences seen in the other two (TAP and HTPc) might have been linked to the different assumptions made, such as burning cane waste and producing biogas (Ghani and Gheewala 2018).

As for the ethanol produced from agave, we reported a carbon footprint of 0.70 kg CO_2_-eq/MJ, whose value was lower than that reported for sugarcane juice, as shown in Table 8. Considering the agave plant-to-bioethanol production chain, the main stage that contributed to the high environmental impact was producing bioethanol from agave juice. This was mainly attributed to energy consumption on purifying the bioethanol. This stage is known to be one of the main hotspots within the life cycle (Sanchez et al. 2021). However, Yan et al. (2011) reported that crop cultivation was the highest contributing factor to environmental impact in terms of GHG. Furthermore, they reported overall GHG emissions of 0.0044 kg CO_2_-eq/MJ whose value was lower than that reported in this study (i.e., 0.70 kg CO_2_-eq/MJ), and our value was higher due to the energy consumed from the Mexican grid.

### Economic analysis

The parameters considered for carrying out the preliminary economic analysis were as follows: installation capacity of 1000 kg/h of raw material, operating time for the plant of 8000 h/year, total operating time of 15 years, and 50% of total costs would be invested in year zero. The inflation rate was 3.8%, the tax rate was 30%, and the depreciation coefficient was 7% (FinancialredMéxico 2018; IPC 2018).

Table 10 shows the costs of equipment and utilities. Table 11 shows a summary of fixed capital, direct production costs, and sales of bioethanol produced from molasses and agave juice.

**Table 10:** Results for immobilized, direct production costs and sales.

		Sugarcane molasses	Agave juice
Raw materials			
Electricity	€/kWh	0.06	
Water	€/m^3^	0.60	
Urea	€/kg	18.0	
Ammonium sulfate	€/ton	357.0	
Magnesium sulfate	€/ton	300.0	
Immobilized			
Major purchased equipment (E)	€	208,400	200,800
Installation costs (M)—60% E	€	125,040	120,480
Buildings—28%	€	35,011	33,734
Piping—45%	€	56,268	54,216
Instrumentation and control—10%	€	12,504	12,048
Electrical—10%	€	12,504	12,048
Insulation—5%	€	6252	6024
Painting—2%	€	2501	2410
Detail engineering—15% (E+M)	€	50,016	48,182
Process engineering, licensing—20%	€	66,688	64,256
Construction—50% (E+M)	€	166,720	160,640
Construction supervision—10% (E+M)	€	33,344	32,128
Total area of process inside battery limit	€	650,208	626,496
Auxiliary service—4% ISBL	€	26,008	25,060
Construction expenses—8% ISBL	€	572,017	50,120
Starting up cost—3.5% ISBL	€	22,757	21,927
Contingency—3.5% ISBL	€	109,235	105,251
Total costs	€	860,225	828,854
Direct production costs			
Total utilities costs	€/year	765,000	467,300
Total cost of labor (6 workers)	€/year	90,000	90,000
Indirect labor	€/year	27,000	27,000
Maintenance	€/year	25,807	24,866
Operating supplies	€/year	43,011	41,443
Laboratory	€/year	18,000	18,000
Payroll changes	€/year	22,500	22,500
Tax	€/year	43,011	41,443
Total costs	€/year	1,034,330	723,323
Sales			
Bioethanol	€/year	1,020,000	270,000
Vinasse	€/year	216,000	198,000
Total sales	€/year	1,236,000	468,200
Economic parameters			
Capital investment	€	1,075,281	1,036,068
Fixed capital	€	860,225	828,854
Working capital	€	215,056	207,214

*E* major purchased equipment, *M* installation costs, *ISBL* total area of process inside battery limit

**Table 11:** Evolution of NPV, IRR, and payback as a function of energy source

		Grid energy vs. renewable energy (%)	NPV (€)	Payback (years)	IRR (%)
Sugarcane molasses					
Basis scenario		100 % vs. 0%	−1,521,947	-	-
Scenario 1		75 % vs. 25 %	−85,967	-	-
Scenario 2		50 % vs. 50 %	1,350,012	6	20
Scenario 3		25 % vs. 75 %	2,785,992	5	31
Scenario 4		0 % vs. 100 %	4,221,972	4	40
Scenario 5		73.5% vs. 26.5%	0	11	7
Agave juice					
Basis scenario		100 % vs. 0%	−1,785,235	-	-
Scenario 1		75 % vs. 25 %	−1,246,743	-	-
Scenario 2		50 % vs. 50 %	−708,250	-	-
Scenario 3		25 % vs. 75 %	−169,758	-	-
Scenario 4		0 % vs. 100 %	368,734	9	11
Scenario 5		17.1% vs. 82.9 %	0	11	9

Equipment costs of the biorefinery were provided by the Aspen Plus® economic package, and the storage tank in this study was to scale. Also, working capital was the raw material stock for 10 days of production. The bioethanol production plant was assumed to be located in the same place as the agave sugar/juice extraction plant (Veracruz, Mexico), whereby the cost of the raw material was assumed to be zero. In addition, Table 11 shows prices for electricity, water, urea, ammonia sulfate, and magnesium sulfate (Budimir et al. 2011; CFE 2019; CONAGUA 2019; SENER 2018). Moreover, it was assumed that six workers, on an annual salary of 15,000 €/worker, were needed to operate the plant.

Furthermore, Table 11 shows that capital investment, fixed capital, and working capital for producing bioethanol from molasses were 1,075,281 €, 860,225 €, and 215,056 €, respectively, while for agave juice these figures were 1,036,068 €, 828,854 €, and 207,214 €, respectively.

On analyzing the data provided by the Aspen Plus simulations, it was observed that from 1000 kg/h of molasses, 170 kg/h of bioethanol and 1080 kg/h of vinasse were produced. In comparison, from 1000 kg/h of agave juice, 45 kg/h of bioethanol and 990 kg/h of vinasse were produced. The vinasse obtained could not be directly applied to the field, although it could be used in conjunction with other residues from the sugar refinery, and in this way, it could be sold (Consorcio 2012). Both products were put on the market, with the following assumptions on price: 0.75 €/kg for bioethanol and 0.025 €/kg for vinasse (biocompost price) (Castañeda-Ayarza and Cortez 2017, Consorcio 2012).

The results obtained from this economic evaluation indicated that neither of the two-bioethanol production scenarios were profitable given that the VPN values obtained were negative (−1,521,947 € for molasses and −1,785,235 € for agave juice), and the time for seeing a return on investment was over 15 years. This might have been mainly due to the high amount of energy used to produce bioethanol which entailed high utility costs. In this study, all energy was assumed to be sourced from the grid, with 1600 kW used for sugarcane and 600 kW for agave.

Therefore, a sensitivity analysis was carried out in order to evaluate how reliable the project would be if part of the grid energy were replaced by renewable energy, assuming that the latter would cost zero because it would be generated at the plant itself. Energy percentages considered in the sensitivity analysis were the following (Table 12):

**Table 12:** Sensitivity analysis for the production of bioethanol from sugarcane molasses and agave juice

Impact category	Unit	100% grid electricity	75% grid electricity	50% grid electricity	25% grid electricity	0% grid electricity
Bioethanol production (molasses)						
GWP	kg CO_2_ eq	3.99E-01	3.50E-01	3.00E-01	2.51E-01	2.01E-01
OPD	kg CFC11 eq	1.31E-06	1.24E-06	1.17E-06	1.10E-06	1.04E-06
HOFP	kg NO_x_ eq	4.83E-04	4.02E-04	3.21E-04	2.40E-04	1.59E-04
EOFP	kg NO_x_ eq	4.92E-04	4.10E-04	3.28E-04	2.45E-04	1.63E-04
TAP	kg SO_2_ eq	1.43E-03	1.27E-03	1.10E-03	9.38E-04	7.73E-04
FEP	kg P eq	1.74E-05	1.51E-05	1.28E-05	1.05E-05	8.17E-06
HTPc	kg 1,4-DCB	5.95E-04	4.98E-04	4.02E-04	3.05E-04	2.08E-04
HTPnc	kg 1,4-DCB	1.49E-02	1.38E-02	1.28E-02	1.17E-02	1.06E-02
FFP	kg oil eq	8.97E-02	7.43E-02	5.89E-02	4.34E-02	2.80E-02
WCP	m^3^	2.90E-02	2.89E-02	2.88E-02	2.87E-02	2.86E-02
Bioethanol production (juice)						
GWP	kg CO_2_ eq	4.83E-01	4.33E-01	3.32E-01	2.56E-01	1.81E-01
OPD	kg CFC11 eq	6.66E-07	5.98E-07	4.60E-07	3.58E-07	2.55E-07
HOFP	kg NO_x_ eq	5.60E-04	4.79E-04	3.13E-04	1.90E-04	6.62E-05
EOFP	kg NO_x_ eq	5.71E-04	4.89E-04	3.20E-04	1.95E-04	6.94E-05
TAP	kg SO_2_ eq	1.17E-03	1.01E-03	6.68E-04	4.17E-04	1.66E-04
FEP	kg P eq	2.15E-05	1.92E-05	1.44E-05	1.09E-05	7.40E-06
HTPc	kg 1,4-DCB	8.65E-04	7.69E-04	5.71E-04	4.24E-04	2.76E-04
HTPnc	kg 1,4-DCB	1.53E-02	1.43E-02	1.21E-02	1.05E-02	8.87E-03
FFP	kg oil eq	1.05E-01	8.99E-02	5.83E-02	3.48E-02	1.13E-02
WCP	m^3^	1.51E-02	1.50E-02	1.48E-02	1.47E-02	1.45E-02

Base scenario: 100% grid energy and 0% renewable energy

Scenario 1: 75% grid energy and 25% renewable energy

Scenario 2: 50% grid energy and 50% renewable energy

Scenario 3: 25% grid energy and 75% renewable energy

Scenario 4: 0% grid energy and 100% renewable energy

Scenario 5: NPV=0

The sensitivity analysis showed that varying the energy source had a significant influence on all three economic parameters (Table 12). On analyzing the results, it was observed that if part of the grid energy were replaced with renewable energy, the two bioethanol production processes would become more economically viable. However, there were considerable differences between both scenarios as molasses were more profitable. So, producing bioethanol was only profitable with the ratios 17.1% grid energy and 82.9% renewable energy and 73.5% grid energy and 26.5% renewable energy for agave juice and molasses, respectively. These considerable differences between both scenarios could be attributed to the lower yields for agave juice in comparison to those for molasses. Hence, producing bioethanol from sugarcane molasses and agave juice was economically viable, and better results were achieved with the former.

## Conclusions

This research aims to compare the environmental and economic performance of using sugarcane juice and agave juice as feedstocks to produce bioethanol in Mexico. On the one hand, producing bioethanol from agave juice had a less environmental impact than sugarcane juice. This was ascribed to the low consumption of pesticides, coal, and water throughout the whole chain. Among stages, bioethanol production contributed to a higher extent (>60%) than cultivation and juice extraction due to the low amounts of ethanol yielded in fermentation. On the other hand, the economic analysis revealed that neither of the feedstocks is feasible if the current Mexican energy grid is employed. However, if 26.5% of renewable energy is employed along the grid, then producing bioethanol from agave juice would be economically feasible. Briefly, using agave juice, rather than sugarcane molasses as a feedstock for producing bioethanol, seems to be more promising from an environmental and economic point of view. On a final note, in Mexico it would be worthwhile creating robust policies to encourage the adoption of renewable energy.

## Data Availability

All data generated and analyzed during this study have been included in this published article.
